# Identifying patients at high risk for carbapenem-resistant Enterobacterales carriage upon admission to acute-care hospitals

**DOI:** 10.1017/ash.2023.339

**Published:** 2023-09-29

**Authors:** Jessica Howard-Anderson, Radhika Prakash Asrani, Chris Bower, Chad Robichaux, Rishi Kamaleswaran, Jesse Jacob, Scott Fridkin

## Abstract

**Background:** Prompt identification of patients colonized or infected with carbapenem-resistant Enterobacterales (CRE) upon admission can help ensure rapid initiation of infection prevention measures and may reduce intrafacility transmission of CRE. The Chicago CDC Prevention Epicenters Program previously created a CRE prediction model using state-wide public health data (doi: 10.1093/ofid/ofz483). We evaluated how well a similar model performed using data from a single academic healthcare system in Atlanta, Georgia, and we sought to determine whether including additional variables improved performance. **Methods:** We performed a case–control study using electronic medical record data. We defined cases as adult encounters to acute-care hospitals in a 4-hospital academic healthcare system from January 1, 2014, to December 31, 2021, with CRE identified from a clinical culture within the first 3 hospital days. Only the first qualifying encounter per patient was included. We frequency matched cases to control admissions (no CRE identified) from the same hospital and year. Using multivariable logistic regression, we compared 2 models. The “public health model” included 4 variables from the Chicago Epicenters model (age, number of hospitalizations in the prior 365 days, mean length of stay in hospitalizations in the prior 365 days, and hospital admission with an infection diagnosis in the prior 365 days). The “healthcare system model” added 4 additional variables (admission to the ICU in the prior 365 days, malignancy diagnosis, Elixhauser score and inpatient antibiotic days of therapy in the prior 365 days) to the public health model. We used billing codes to determine Elixhauser score, malignancy status, and recent infection diagnoses. We compared model performance using the area under the receiver operating curve (AUC). **Results:** We identified 105 cases and 441,460 controls (Table 1). CRE was most frequently identified in urine cultures (46%). All 4 variables included in the public health model and the 4 additional variables in the healthcare system model were all significantly associated with being a case in unadjusted analyses (Table 1). The AUC for the public health model was 0.76, and the AUC for the healthcare system model was 0.79 (Table 2; Fig. 1). In both models, a prior admission with an infection diagnosis was the most significant risk factor. **Conclusions:** A modified CRE prediction model developed using public health data and focused on prior healthcare exposures performed reasonably well when applied to a different academic healthcare system. The addition of variables accessible in large healthcare networks did not meaningfully improve model discrimination.

**Figure:**
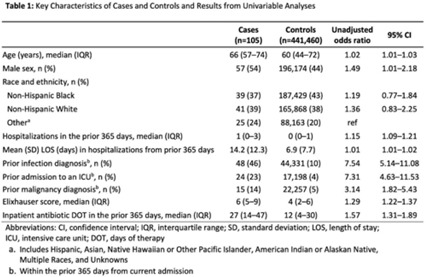

**Figure:**
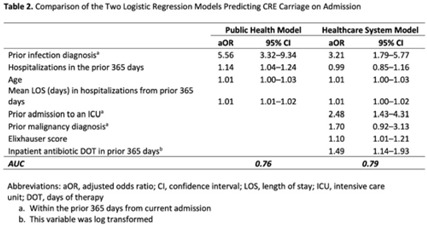

**Figure:**
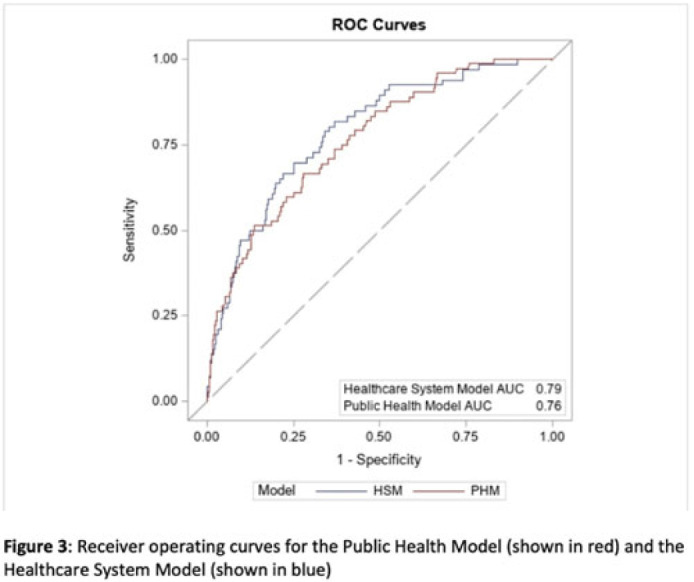

**Disclosures:** None

